# The Avon Longitudinal Study of Parents and Children (ALSPAC): an update on the enrolled sample of index children (the G1 cohort) in 2025

**DOI:** 10.12688/wellcomeopenres.25018.2

**Published:** 2026-04-19

**Authors:** Kate Northstone, Elizabeth Brierley, Jess Harvey, Sarah Matthews, Mark Mummé, Rich Hobbs

**Affiliations:** 1 Population Health Sciences, University of Bristol Medical School, Bristol, England, UK

**Keywords:** ALSPAC, Children of the 90s, birth cohort study, cohort profile, enrolment

## Abstract

The Avon Longitudinal Study of Parents and Children (ALSPAC) is an ongoing prospective population-based study that has been running for almost 35 years. Pregnant women with expected dates of delivery falling between 1 ^st^ April 1991 and 31 ^st^ December 1992 were recruited and the health and development of the index children from these pregnancies and that of their family members have been followed ever since. At the time of enrolment, the sampling frame for those eligible to enrol was constructed retrospectively using linked recruitment and health service records. Further explicit recruitment drives took place at the ages of 7 and 18 years to enrol G1 participants (the index children) that were eligible but did not take part originally. These were supplemented by opportunistic contacts from the age of 7 resulting in additional participants being enrolled. Around the age of 30, a further concerted effort was undertaken to encourage unenrolled but eligible participants to take part in the study’s ‘@30’ clinic. This data note updates the status of recruitment of the index children in 2025, following the completion of the clinic. In total, 1014 additional G1 participants have been enrolled in the study since the age of 7 years, with 101 of these in the most recent phase of recruitment that has taken place since the age of 24. At the time of writing ALSPAC now has a baseline cohort of 15,002 G1 participants alive at 1 year of age.

## Introduction

The Avon Longitudinal Study of Parents and Children (ALSPAC) is a birth cohort study that recruited pregnant women with an estimated date of delivery between April 1991 and December 1992 who were resident in and around the city of Bristol in the South West of England https://wellcomeopenresearch.org/articles/3-34/v2. ^1^ ^,^ ^2^ Between (1990 and 1992) a total of 14,541 pregnancies were enrolled (some women had more than one eligible pregnancy during this period) and form the baseline cohort. The parents (Generation 0: ‘G0’), the children (Generation 1: ‘G1’:) and now their children (Generation 2: ‘G2’), have been followed ever since. ALSPAC is one of the most richly phenotyped studies of its kind in the world. Data has been collected via questionnaires, face to face visits (called ‘Focus’ clinics) which has included the collection of biological samples and through linkage to routine administrative data. The study website contains details of all the data that is available through a fully searchable data dictionary and variable search tool ( http://www.bristol.ac.uk/alspac/researchers/our-data/).

We have previously described the original cohort of families and their follow up until G1 reached the age of 18 years; ^1^ ^,^ ^2^ this includes details on how recruitment was subsequently extended to include those families who could have taken part according to the original eligibility criteria but did not initially enrol. *Phase I* recruitment which took place between 1990 and 1992, enrolled women during pregnancy and shortly after birth. We have also previously described subsequent recruitment drives, known as *Phase II, III and IV* enrolment up until the age of 24 years. ^3^ Here we provide an update on *phase V* recruitment – this phase enrolled G1 participants from the age of 24, up to and including the 30-year face to face assessment that took place between 2021 and 2024. We therefore refer to phase V as 30+, it should be noted that participants enrolled as part of phase V may have been older than 30 at the time they enrolled.

## Methods

Details on previous recruitment efforts are described in the second version of the cohort profile. ^3^ As reported there, ALSPAC continues to welcome any opportunistic contacts with potential participants who were eligible but had not previously enrolled. This was intensified in the build up to and during the ‘@30’ face to face assessment where it was important to optimise the number of participants we saw, particularly as they were approaching peak age of fertility and we were actively enrolling G2 participants into the study.

There were three routes through which new participants could join the study during *Phase V.*

1. Re-engagement packs: To encourage enrolment, participants we had lost contact with, as well as those not previously enrolled, were invited to re-join or enrol in the study and were provided with a £20 incentive.
2. Enrolment of the second generation (G2): Eligible individuals who were pregnant or already had children were recruited into the study via their pregnancy or child (ren).
3. Ad hoc approaches: Enrolment that occurred outside of the two methods above, such as potential participant reaching out to the study after a piece appeared in the local media.

### Study numbers

As previously reported, ^3^ a total of 20,248 G0 pregnancies (resulting in 20,505 potential G1 participants) were eligible to take part in the study. At the fetal stage, i.e. in *phase I*, the G0 mothers of 14,676 G1 participants enrolled. During *phase II*, 456 G1 participants enrolled through the systematic campaign at age 7 and *phase III* saw an additional 262 G1 participants enrol via opportunistic contacts between the ages of 8 and 18. In *phase IV*, 195 additional G1 participants enrolled between the ages of 18 and 24 years.

**Figure 1.**
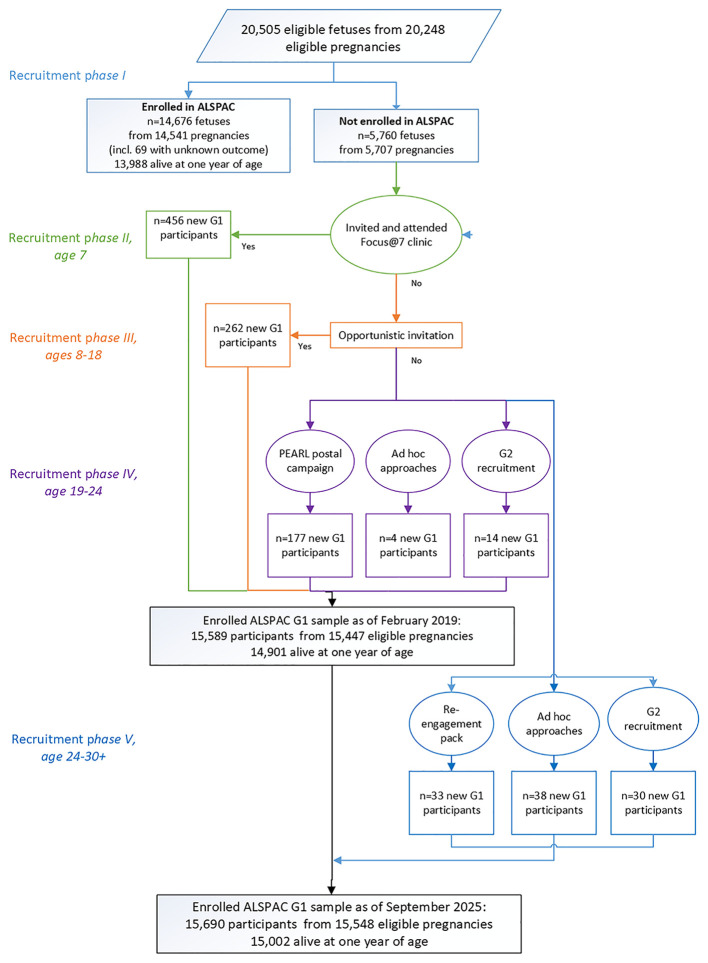
The ALSPAC enrolment campaign flow diagram, illustrating enrolment phases I to V. (adapted from Boyd *et al*., 2013 ^1^; Oxford University Press, Creative Commons Attribution Non-Commercial License).

A further 101 participants have joined the study since the age of 24, i.e. enrolled through *phase V.* Of these, 33 participants were recruited via a re-engagement pack, 30 through enrolling their own pregnancy and a further 38 via ad hoc approaches ( Figure 1). It should be noted that at the time of writing, *phase V* participants may not have contributed any data to the resource.

In summary therefore, a total of 1014 G1 participants have enrolled who were not in the initial study cohort (456, 262, 195 and 101 recruited during *Phases II, III*, *IV* and *V* respectively). The total cohort for analyses from September 2025, is 15,548 pregnancies, resulting in 15,690 foetuses with known outcome. Of this total sample of 15,690 potential G1 participants, 15,002 were alive at 1 year of age and are considered the baseline cohort for reporting purposes. It is important to note that data accessed by researchers does not include that provided for triplet and quadruplet pregnancies. This means that there are 10 G1 participants from those alive at one year of age who will not appear in tabulations run by researchers. We ask users of the resource to quote the figures presented here and in our publications checklist ( https://www.bristol.ac.uk/media-library/sites/alspac/documents/alspac-publications-checklist.pdf) when describing the available baseline sample.

Table 1 summarises the G1 cohort and provides illustrative examples of the potential data available at the time of writing – it is important to note these can change at any time, primarily due to participant consents but also to new data being generated all the time.

**Table 1.** Activity of G1 (the cohort of index children) and illustrative data availability in ALSPAC, from the baseline cohort of 15,002 participants alive at one year of age.

G1 Activity as of *September 2025*	N (%)
Not died or withdrawn from the study	13,397 (89.3%)
Current known address (email and/or postal)	10,393 (69.3%)
Linked to Primary health care data	11,810 (78.7%)
Linked to Secondary health care data	12,700 (84.7%)
At least one DXA * measure between the ages of 7 and 30	9,263 (61.7%)
Genomics data 2	8,952 (59.7%)
Methylation at multiple time points	1,003 (6.7%)
G2 parent	1,985 (13.2%)

[i]* Body composition assessed using Dual-energy X-ray absorptiometry.

## Cohort profile data file

The variables described in the cohort profile data file (ALSPAC reference: cp_4a at the time of writing) are provided as a matter of course with all data requests (see Table 2). The denominator for this file is the 20,505 G1 eligible sample previously described. However, the total number of cases supplied in any G1 data request is 15,690, representing all foetuses with known outcome. This will include participants who have formally withdrawn from the study or who are at high risk of disclosure, however, their data will be supressed. This is in addition to the triplets and quadruplets noted above. Such suppressions will be labelled in all datasets. It is possible that those participants who have enrolled in the study in later phases may differ from those who were originally enrolled at the start, particularly in terms of demographics. They are more likely to drop out of complex complete-case analyses as they will not have data that was collected earlier on in the study. Nevertheless, researchers may want to address potential issues of selection bias by considering phase of recruitment in their analyses. The variables described in Table 2 will enable researchers to determine their own analytical cohort. For example, a research study examining an outcome at 3 years of age would likely want to remove later phase recruitment from their analyses (keep those where “in_core”=Yes) and restrict to those who are alive at one year of age (“kz011b”=Yes), whilst those interested in an outcome assessed at 17 years of age would keep those recruited up to and including Phase 3 (“in_core”=Yes, “in_phase2=Yes” or “in_phase3”=Yes).

**Table 2.** Cohort Profile Data File (Version 4a at the time of writing; variables have not changed from version 3a).

Variable name	Variable Label
in_alsp	Enrolled in ALSPAC
in_core	Enrolled as part of original core sample, Phase I
in_phase2	Enrolled as part of phase II, during focus@7
in_phase3	Enrolled as part of phase III, after focus@7 up to age 18
in_phase4	Enrolled as part of phase IV, >=19 & <=24
in_phase5	Enrolled as part of phase V, after F24 up to @30
tripquad	Participant is a triplet or quad
kz011b	Participant was alive at 1 year of age
kz021	Participant sex

If you have any questions about the data or how to access it, please email alspac-data@bristol.ac.uk.

## Consent

Ethical approval for the study was obtained from the ALSPAC Ethics and Law Committee (ALEC; IRB00003312) and the Local Research Ethics Committees. Informed consent for the use of all data collected was obtained from participants following the recommendations of the ALSPAC Ethics and Law Committee at the time – this may have been verbal or written depending on the data collected. Participants can contact the study team at any time to retrospectively withdraw consent for their data to be used. Study participation is voluntary and during all data collection sweeps, information was provided on the intended use of data. Further details are available online: http://www.bristol.ac.uk/alspac/researchers/research-ethics/.

## Data Availability

ALSPAC data access is through a system of managed open access. The steps below highlight how to apply for access to the data included in this data note and all other ALSPAC data:
1. Please read the ALSPAC access policy ( http://www.bristol.ac.uk/media-library/sites/alspac/documents/researchers/data-access/ALSPAC_Access_Policy.pdf) which describes the process of accessing the data and samples in detail, and outlines the costs associated with doing so. 2. You may also find it useful to browse our fully searchable research proposals database ( https://proposals.epi.bristol.ac.uk/?q=proposalSummaries), which lists all research projects that have been approved since April 2011. 3. Please submit your research proposal ( https://proposals.epi.bristol.ac.uk/) for consideration by the ALSPAC Executive Committee. You will receive a response within 10 working days to advise you whether your proposal has been approved.
