# Supplementary material for: The Avon Longitudinal Study of Parents and Children (ALSPAC): an update on the enrolled sample of index children (the G1 cohort) in 2025
Source: Wellcome Open Res. 2026 Apr 19;10:689. Originally published 2025 Dec 22. [Version 2] doi: 10.12688/wellcomeopenres.25018.2 (PMC13146456; doi:10.12688/wellcomeopenres.25018.2)
Supplement: Peer review history

# Reviewer report: Reviewer response for version 2

**DOI:** 10.21956/wellcomeopenres.29127.r153777

**Published:** 2026-05-05

**Authors:** Sivenesi Subramoney

**Affiliations:** 1 Virginia Commonwealth University Department of Psychology (Ringgold ID: 218033), Richmond, Virginia, USA; 2 University of California Merced, Merced, California, USA

Thank you for inviting me to review this data note. The data note provides an update on the enrolment of participants to the ALSPAC, an ongoing prospective population-based study. The data note is very clearly written and provides a good overview of the recruitment for the ‘@30’ clinic. I have a few minor comments”

**Methods**

 “This was intensified in the build up to and during the ‘@30’ face to face assessment where it was important to optimise the number of participants we saw, particularly as they were approaching peak age of fertility and we were actively enrolling G2 participants into the study.”

 Approximate how long was this build up period during which recruitment efforts were intensified?

 “Enrolment that occurred outside of the two methods above, such as potential participant reaching out to the study after a piece appeared in the local media.”

 It think it will be helpful to clarify -were there advertisements inviting potential participants to join the study or were these media appearances write-ups/features on the study and its findings.

 “2. Enrolment of the second generation (G2): Eligible individuals who were pregnant or already had children were recruited into the study via their pregnancy or child (ren).”

 How did individuals know that it was possible to still be recruited into the study - do participants receive some correspondence stating that enroling more recent births into the study is an ongoing possibility?

 “It is important to note that data accessed by researchers does not include that provided for triplet and quadruplet pregnancies.”

 This sentence is somewhat difficult to understand, consider rephrasing to improve the clarity and readability of the data note.

 The Cohort profile data file and Consent sections are detailed, clear, and easy to follow.

Are sufficient details of methods and materials provided to allow replication by others?

Yes

Is the rationale for creating the dataset(s) clearly described?

Yes

Are the datasets clearly presented in a useable and accessible format?

Yes

Are the protocols appropriate and is the work technically sound?

Yes

Reviewer Expertise:

Developmental Psychologist with experience in maternal and child health

I confirm that I have read this submission and believe that I have an appropriate level of expertise to confirm that it is of an acceptable scientific standard.

# Reviewer report: Reviewer response for version 2

**DOI:** 10.21956/wellcomeopenres.29127.r153639

**Published:** 2026-04-23

**Authors:** Gillian Santorelli

**Affiliations:** 1 Bradford Teaching Hospitals NHS Foundation Trust, Bradford, England, UK

Thank you to the authors for revising their manuscript.

Are sufficient details of methods and materials provided to allow replication by others?

Partly

Is the rationale for creating the dataset(s) clearly described?

Yes

Are the datasets clearly presented in a useable and accessible format?

Yes

Are the protocols appropriate and is the work technically sound?

Yes

Reviewer Expertise:

Epidemiology, longitudinal cohort studies, statistical modelling, maternal and child health, childhood obesity and cardiometabolic health.

I confirm that I have read this submission and believe that I have an appropriate level of expertise to confirm that it is of an acceptable scientific standard.

# Reviewer report: Reviewer response for version 1

**DOI:** 10.21956/wellcomeopenres.27564.r149451

**Published:** 2026-03-25

**Authors:** Abdul Wajid

**Affiliations:** 1 University of Calgary, Calgary, Alberta, Canada

Hi, I am thankful for inviting me to review this data note. I found this update a thorough work and effective presentation by the researchers/authors. After reviewing it, I have some comments, please see below; all are major and should be addressed.

 1. For phase V recruitment, the authors mention age range as 24, up to and including 30-year. As the original recruitment for this cohort was started in 1991/92 with some even delivered moms. By 2024 the age of a G1 participant (an index child) could go beyond 30 years, up to 32 years. Unless, there was an explicit reason for not to recruit participants beyond 30 years, the statement should not restrict at 30 years but should clearly indicate beyond 30 year. Interestingly, the Figure 1 shows age range for phase V as 24-30+. 

 2. For phase V recruitment, three routes were chosen; however, only re-engagement packs, the route 1, participants were provided with monetary incentive. Was there a specific reason for not incentivizing other two groups and only providing incentives to this group?

Are sufficient details of methods and materials provided to allow replication by others?

Partly

Is the rationale for creating the dataset(s) clearly described?

Partly

Are the datasets clearly presented in a useable and accessible format?

No

Are the protocols appropriate and is the work technically sound?

Partly

Reviewer Expertise:

I am an epidemiologist, with interest in more than one areas - reproductive health, perinatal, maternal and child health, machine learning/data science.

I confirm that I have read this submission and believe that I have an appropriate level of expertise to confirm that it is of an acceptable scientific standard, however I have significant reservations, as outlined above.

# Response

**Published:** 2026-04-07

**Authors:** Kate Northstone

**Affiliations:** University of Bristol, UK

Thank you so much for your helpful comments. We repat your comments below and add our response in bold:  

 1. For phase V recruitment, the authors mention age range as 24, up to and including 30-year. As the original recruitment for this cohort was started in 1991/92 with some even delivered moms. By 2024 the age of a G1 participant (an index child) could go beyond 30 years, up to 32 years. Unless, there was an explicit reason for not to recruit participants beyond 30 years, the statement should not restrict at 30 years but should clearly indicate beyond 30 year. Interestingly, the Figure 1 shows age range for phase V as 24-30+. 

**Thank you for this comment. You are correct that the participants could be older than 30 at the time of phase V recruitment. However, we are referring to the specific data collection points. The @30 clinic was the key driver for phase V recruitment and that is why we refer to 30+, as new  participants could indeed be older than 30 when they enrolled. We have added a sentence to the end of the introduction which clarifies this.**

 2. For phase V recruitment, three routes were chosen; however, only re-engagement packs, the route 1, participants were provided with monetary incentive. Was there a specific reason for not incentivizing other two groups and only providing incentives to this group?

**We did not have ethical approval to incentivise the other two groups and as they approached us voluntary for both of these routes we did not feel an incentive was necessary.**

# Reviewer report: Reviewer response for version 1

**DOI:** 10.21956/wellcomeopenres.27564.r149446

**Published:** 2026-03-14

**Authors:** Gillian Santorelli

**Affiliations:** 1 Bradford Teaching Hospitals NHS Foundation Trust, Bradford, England, UK

This data note updates the enrolment status of the ALSPAC G1 cohort up to 2025, including Phase V recruitment and the ‘@30’ clinic. It serves as a helpful reference for researchers when defining ALSPAC denominators and analytic samples. I found the note clear and well-structured but recommend minor revisions to sharpen the definition of key denominators and provide slightly more explicit guidance for users.​

**Major comments**

**Standardising denominators: **The paper uses several related terms (“baseline cohort”, “baseline sample”, “total sample size for analyses”, and the denominator for the cohort profile data file). For readers less familiar with ALSPAC, it would be helpful to standardise terminology and present a concise hierarchy of denominators early in the “Study numbers” section (e.g., total eligible G1; G1 with known outcome; G1 alive at 1 year; analytic baseline available to researchers). At present, the abstract refers to “a baseline sample of 14,992 G1 participants alive at 1 year of age”, whereas later text notes that 15,002 were alive at one year and that 10 triplet/quad G1 are not present in researcher datasets. Making the relationship between these figures explicit, and using consistent labels throughout, would reduce confusion and avoid misreporting in downstream publications.​ Explicitly documenting these denominators will also help ensure consistency in how ALSPAC samples are described across publications.

**Addressing potential selection bias: **The description of the recruitment phases across Phases I–V is clear, and the breakdown of Phase V routes is useful. However, the note does not currently discuss the potential for selection bias among later enrolees.​ I recommend adding a short paragraph acknowledging that later phases of recruitment may introduce additional selection (e.g., those who are now parents, more engaged participants), and suggesting that analysts consider phase of enrolment in sensitivity analyses or as an adjustment variable. This would provide a useful steer for data users without requiring extensive new analyses.​

**Guidance on use of cp_4a and phase variables: **Table 2 provides a helpful summary of the variables in the cohort profile data file (cp_4a). Adding a brief statement describing typical analytic uses for these variables would be particularly useful for new users. For example, using kz011b to define the subset alive at one year; the in_phase indicators to restrict to particular enrolment phases or to stratify analyses.​ A short illustrative example (e.g., restricting to certain phases for longitudinal analyses with early-life measures) would further support consistent practice.

**Clarify Table 1: **Table 1 (“Activity of G1 and illustrative data availability”) is very useful but could be clearer. First, the denominator for the counts (e.g. 13,397 “not died or withdrawn”, 10,393 with known address) is not explicitly  stated. Add a footnote specifying the denominator (e.g,. 15,002 G1 alive at 1 year, or 14,992 if that is the analytic baseline) and consider adding percentages alongside counts, which would help readers quickly gauge coverage of linkage and clinic data.​ Second, the different measures of “contactability” are confusing as currently presented. The number of participants to whom questionnaires can be sent (11,096) appears to exceed the number with a known address (10,393), which is puzzling if these categories are assumed to be nested. It would be helpful to briefly clarify how these categories are defined and whether they reflect different modes of contact (e.g. postal address, email-only contact, or other routes). A short explanatory note or table footnote would make this clear. Finally, Table 1 includes examples of biological data available in ALSPAC (e.g. DXA measures, genomics, methylation). These examples are helpful but might be interpreted as a comprehensive list. Given the breadth of ALSPAC’s molecular data (e.g. metabolomics and other omics platforms at multiple time points), you could clarify that the table provides illustrative examples rather than an exhaustive list, and explicitly direct readers to the data dictionary or other documentation for a full description of available omics resources.

**Minor comments**

**Typographical and wording issues: **In the description of ad hoc enrolment, there is a small typographical error (“such a as”) that should be corrected.​

**Phase V data contribution: **The paper notes that Phase V participants “may not have contributed any data to the resource” at the time of writing. It would be helpful to be slightly more explicit about which types of data are expected for Phase V participants (e.g. @30 clinic measures, questionnaires, linkage only), and to recommend that analysts check data completeness by phase when defining analytic samples, particularly if their work relies on clinical measures or repeated assessments.​

**Data suppression and discrepancies in counts: **It may be useful to briefly acknowledge that some participants included in headline counts will have data suppressed in researcher datasets (e.g. those at high disclosure risk or who have withdrawn). A short sentence explaining that user counts may therefore occasionally diverge from the reported totals would complement existing guidance to cite these denominators and the publication checklists.

 Addressing the points would further strengthen the note as a reference for researchers working the ALSPAC data.

Are sufficient details of methods and materials provided to allow replication by others?

Partly

Is the rationale for creating the dataset(s) clearly described?

Yes

Are the datasets clearly presented in a useable and accessible format?

Yes

Are the protocols appropriate and is the work technically sound?

Yes

Reviewer Expertise:

Epidemiology, longitudinal cohort studies, statistical modelling, maternal and child health, childhood obesity and cardiometabolic health.

I confirm that I have read this submission and believe that I have an appropriate level of expertise to confirm that it is of an acceptable scientific standard, however I have significant reservations, as outlined above.

# Response

**Published:** 2026-04-07

**Authors:** Kate Northstone

**Affiliations:** University of Bristol, UK

**We thank the reviewer for their valuable comments. We repeat their comments below and respond in bold.**

 Major comments

 Standardising denominators: The paper uses several related terms (“baseline cohort”, “baseline sample”, “total sample size for analyses”, and the denominator for the cohort profile data file). For readers less familiar with ALSPAC, it would be helpful to standardise terminology and present a concise hierarchy of denominators early in the “Study numbers” section (e.g., total eligible G1; G1 with known outcome; G1 alive at 1 year; analytic baseline available to researchers). At present, the abstract refers to “a baseline sample of 14,992 G1 participants alive at 1 year of age”, whereas later text notes that 15,002 were alive at one year and that 10 triplet/quad G1 are not present in researcher datasets. Making the relationship between these figures explicit, and using consistent labels throughout, would reduce confusion and avoid misreporting in downstream publications.​ Explicitly documenting these denominators will also help ensure consistency in how ALSPAC samples are described across publications.

**Thank you so much for this valuable comment. It is important that we clearly distinguish between the numbers we have available for analysis compared to the actual numbers enrolled as we receive many queries on this from researchers this is why we now explicitly separate this out in the cohort profile. In line with the reviewer comment, we have now made several changes throughout the paper to clarify this. The abstract now states 15,002 and we refer to a “baseline cohort” of G1s alive at one year of age throughout.  We also make clear  the “original” cohort who were first enrolled in the early 90s**

 Addressing potential selection bias: The description of the recruitment phases across Phases I–V is clear, and the breakdown of Phase V routes is useful. However, the note does not currently discuss the potential for selection bias among later enrolees.​ I recommend adding a short paragraph acknowledging that later phases of recruitment may introduce additional selection (e.g., those who are now parents, more engaged participants), and suggesting that analysts consider phase of enrolment in sensitivity analyses or as an adjustment variable. This would provide a useful steer for data users without requiring extensive new analyses.​ **Thank you – this is a valid point and we have added words to this effect just after the wording about Table 2.**

 Guidance on use of cp_4a and phase variables: Table 2 provides a helpful summary of the variables in the cohort profile data file (cp_4a). Adding a brief statement describing typical analytic uses for these variables would be particularly useful for new users. For example, using kz011b to define the subset alive at one year; the in_phase indicators to restrict to particular enrolment phases or to stratify analyses.​ A short illustrative example (e.g., restricting to certain phases for longitudinal analyses with early-life measures) would further support consistent practice. **Thank you for this useful comment - we have provided some additional info in the text added above.**

 Clarify Table 1: Table 1 (“Activity of G1 and illustrative data availability”) is very useful but could be clearer. First, the denominator for the counts (e.g. 13,397 “not died or withdrawn”, 10,393 with known address) is not explicitly  stated. Add a footnote specifying the denominator (e.g,. 15,002 G1 alive at 1 year, or 14,992 if that is the analytic baseline) and consider adding percentages alongside counts, which would help readers quickly gauge coverage of linkage and clinic data.​ Second, the different measures of “contactability” are confusing as currently presented. The number of participants to whom questionnaires can be sent (11,096) appears to exceed the number with a known address (10,393), which is puzzling if these categories are assumed to be nested. It would be helpful to briefly clarify how these categories are defined and whether they reflect different modes of contact (e.g. postal address, email-only contact, or other routes). A short explanatory note or table footnote would make this clear. Finally, Table 1 includes examples of biological data available in ALSPAC (e.g. DXA measures, genomics, methylation). These examples are helpful but might be interpreted as a comprehensive list. Given the breadth of ALSPAC’s molecular data (e.g. metabolomics and other omics platforms at multiple time points), you could clarify that the table provides illustrative examples rather than an exhaustive list, and explicitly direct readers to the data dictionary or other documentation for a full description of available omics resources.

**Again, really helpful comments. We have added to the title and added percentages as suggested and removed the rows relating to Q and clinic invites to reduce confusion (for the reviewer’s benefit these referred to flags on our administrative database but participants could have been lost to contact after those flags were created). We have also clarified in the text describing Table 1 that these are illustrative.**

 Minor comments

 Typographical and wording issues: In the description of ad hoc enrolment, there is a small typographical error (“such a as”) that should be corrected.​ Corrected

 Phase V data contribution: The paper notes that Phase V participants “may not have contributed any data to the resource” at the time of writing. It would be helpful to be slightly more explicit about which types of data are expected for Phase V participants (e.g. @30 clinic measures, questionnaires, linkage only), and to recommend that analysts check data completeness by phase when defining analytic samples, particularly if their work relies on clinical measures or repeated assessments.​ **The point we are making here is that a participant may have enrolled but not yet provided data – they may have enrolled too late to attend @30 and not yet completed a questionnaire but may have consented to linkage or any combination thereof. As time passes we hope these participants will provide data collected in the future but at this point we don’t know for sure.**

 Data suppression and discrepancies in counts: It may be useful to briefly acknowledge that some participants included in headline counts will have data suppressed in researcher datasets (e.g. those at high disclosure risk or who have withdrawn). A short sentence explaining that user counts may therefore occasionally diverge from the reported totals would complement existing guidance to cite these denominators and the publication checklists. **Thank you we have added words to this effect in the cohort profile data file paragraph.**
